# Microcirculation in Patients with Takotsubo Syndrome—The Prospective CIRCUS-TTS Study

**DOI:** 10.3390/jcm10102127

**Published:** 2021-05-14

**Authors:** Christian Möller, Thomas Stiermaier, Moritz Meusel, Christian Jung, Tobias Graf, Ingo Eitel

**Affiliations:** 1 Department of Cardiology, Angiology, Pulmonology, Nephrology and Intensive Care Medicine, Medical Clinic I, Leopoldina Hospital Schweinfurt, 97422 Schweinfurt, Germany; cmoeller@leopoldina.de; 2 Department of Cardiology, Angiology and Intensive Care Medicine, Medical Clinic II, University Heart Center Lübeck, 23538 Lübeck, Germany; thomas.stiermaier@uksh.de (T.S.); moritz.meusel@uksh.de (M.M.); tobias.graf@uksh.de (T.G.); 3 German Center for Cardiovascular Research (DZHK), Partner Site Hamburg/Kiel/Lübeck, 23538 Lübeck, Germany; 4 Division of Cardiology, Pulmonology and Vascular Medicine, Medical Faculty, University Hospital Düsseldorf, 40225 Düsseldorf, Germany; christian.jung@med.uni-duesseldorf.de

**Keywords:** Takotsubo syndrome, stress cardiomyopathy, pathophysiology, microcirculation, microvascular dysfunction

## Abstract

The pathophysiology of Takotsubo syndrome (TTS) is incompletely understood. A sympathetic overdrive with coronary microvascular dysfunction might play a central role. The aim of our study was to assess the status of the systemic microcirculation (MC) of patients with TTS, compared to patients with myocardial infarction (MI) and healthy subjects. The systemic microvascular function of 22 TTS patients, 20 patients with MI and 20 healthy subjects was assessed via sublingual sidestream dark-field imaging. In TTS and MI patients, measurements were performed during the acute phase (day 1, 3 and 5) and after 3 months. The measurement in healthy subjects was performed once. The assessed parameters were number of vessel crossings, number of perfused vessel crossings, proportion of perfused vessels, total vessel density and perfused vessel density. The results did not show relevant differences between the investigated groups. Some minor, albeit statistically significant, differences occurred rather randomly. The MC parameters of the TTS group did not show any relevant changes in the temporal course. A systemic microvascular dysfunction could not be identified as a contributing factor in the pathogenesis of TTS. A possible microvascular dysfunction might instead be caused by a local effect restricted to the coronary microvascular bed.

## 1. Introduction

Takotsubo syndrome (TTS) is an increasingly recognized acute heart failure syndrome, affecting the left and/or right ventricle. A central feature of the prominent wall motion disturbances is their transient nature. After weeks or months, a complete recovery of the systolic function can be observed [1,2]. The onset of TTS is typically acute und accompanied by the symptoms of an acute coronary syndrome—mostly chest pain and dyspnea. A stressful trigger is frequent, but not always present. The vast majority of the affected patients are postmenopausal women [3]. A satisfying explanation for this observation is still lacking [4]. During the acute presentation, a reliable clinical differentiation from myocardial infarction (MI) is virtually impossible, because both entities show similar clinical, electrocardiographic and laboratory findings [1]. Coronary angiography is required to exclude a culprit coronary lesion which explains the pronounced contraction abnormalities.

The exact pathomechanisms of TTS are still incompletely understood. In the meantime, different pathophysiological hypotheses have been discussed [5,6,7]. While there is the widest consensus that catecholamines and the sympathetic nervous system play a central role, the link between sympathetic overdrive and the contractile dysfunction of the myocardium is still a missing piece in the puzzle. However, there is growing evidence to suggest that a microvascular dysfunction might be responsible for the distinctive wall motion disturbances in TTS. In this scenario, catecholamine-triggered vasospasms in the coronary microvascular bed lead to transient ischemia with consecutive contractile dysfunction of the affected myocardium [6,8]. This transient ischemia leads to prolonged myocardial dysfunction, continuing beyond the period of hypoperfusion in terms of “myocardial stunning” [9]. It is unclear if this effect is systemic or restricted to the coronary microcirculation (MC). However, since elevated levels of catecholamines [10] and endothelin-1 [11]—both possible surrogate parameters for vasoconstriction—have been detected in the systemic circulation of TTS patients, consecutive microvascular dysfunction might have a systemic effect.

The aim of our prospective MicroCIRCUlation in PatientS with TakoTsubo Syndrome (CIRCUS-TTS) study was, therefore, to comprehensively assess the status of systemic MC in TTS patients in the acute phase and after a follow up of 3 months via sublingual sidestream dark-field (SDF) imaging.

## 2. Materials and Methods

### 2.1. Study Population

**Figure 1:**
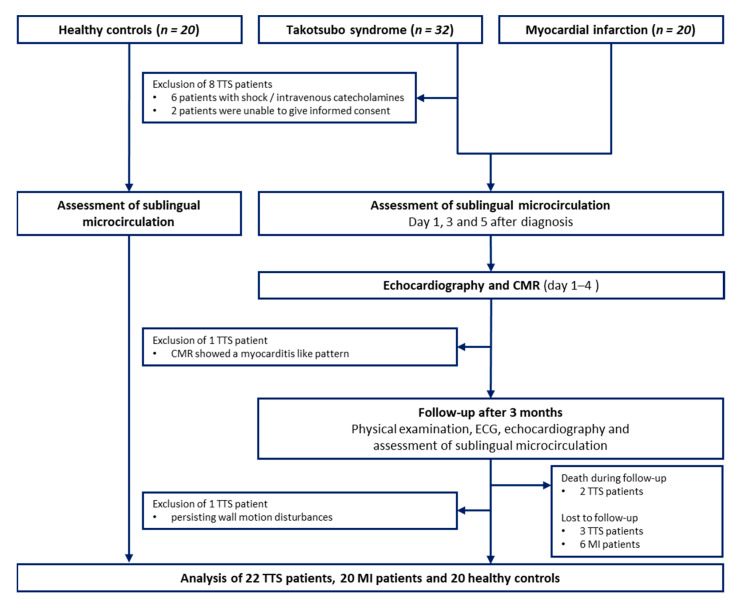
Study flow chart: The investigated groups consisted of prospectively enrolled patients with TTS, age- (+/− 5 years) and gender-matched patients with MI, as well as healthy subjects (without age and gender matching). CMR = cardiovascular magnetic resonance; ECG = electrocardiography; MI = myocardial infarction; TTS = Takotsubo syndrome.

The study was performed between January 2016 and December 2018 at the University Heart Center Lübeck, Department of Cardiology, Angiology and Intensive Care Medicine. A total of 32 TTS patients fulfilling the proposed diagnostic criteria from the Taskforce on TTS of the Heart Failure Association of the European Society of Cardiology [1] were prospectively enrolled. The control groups consisted of 20 prospectively enrolled, age- (+/− 5 years) and gender-matched patients with acute MI (defined according to current guidelines requiring cardiac biomarker elevation and a documented coronary culprit lesion [12]) and 20 unmatched healthy subjects. A detailed overview is provided as a study flow chart in Figure 1.

The exclusion criteria were (1) shock (defined as (a) systolic blood pressure < 90 mmHg for >30 min or vasopressors required to achieve a blood pressure ≥ 90 mmHg; (b) pulmonary congestion or elevated left-ventricular filling pressures; (c) signs of impaired organ perfusion with at least one of the following criteria: altered mental status; cold, clammy skin; oliguria; increased serum-lactate > 2 mmol/L [13]), (2) age < 18 years, (3) patient unable to give informed consent, (4) injuries or malignancies in the oral cavity, and (5) in the case of TTS, detection of significant late gadolinium enhancement in cardiac magnetic resonance imaging in the area of wall motion disturbances. Due to these criteria, 10 TTS patients were subsequently excluded from the study.

### 2.2. Study Protocol

Patients with the symptoms of an acute coronary syndrome, typical electrocardiographic findings and/or elevated myocardial serum markers according to the diagnostic criteria of ST-segment elevation myocardial infarction (STEMI) or Non-ST-segment elevation myocardial infarction (NSTEMI) underwent coronary angiography and left ventriculography. Patients with characteristic wall motion disturbances (apical, midventricular, or basal ballooning) and fulfilling of the aforementioned diagnostic criteria for TTS were included in the TTS-group. In the case of the detection of a culprit coronary lesion, patients were classified as acute MI and were included in the MI group. Additionally, we evaluated a control group of 20 healthy subjects (without age and gender matching).

After patients gave written informed consent, we performed the assessment of systemic microvascular function via sublingual SDF imaging (for detailed description see below) on days 1 (=day of diagnosing TTS/myocardial infarction), 3 and 5 (only one measurement in the healthy control group). Between days 1 and 4, MI and TTS patients underwent transthoracic echocardiography. Additionally, TTS patients underwent cardiac magnetic resonance (CMR) imaging for diagnosis confirmation in the case of missing contraindications (two patients refused the examination, one patient had a cardiac pacemaker which was not CMR-conditional). In cases with missing CMR, diagnosis confirmation was performed through the documentation of a complete recovery of left ventricular systolic function. TTS patients were excluded from the study in the case of the detection of significant late gadolinium enhancement in the area of wall motion disturbances [14]. Furthermore, MI and TTS patients received a thorough physical examination, routine blood sample analysis and electrocardiography. Three months after the acute presentation, we performed a follow-up, including current clinical history, thorough physical examination, transthoracic echocardiography and sublingual SDF imaging (for TTS and MI patients).

The study was conducted according to the principles of the Helsinki Declaration and after approval by the local ethics committee. All patients gave written informed consent.

### 2.3. Sidestream Dark-Field (SDF) Imaging

The assessment of systemic MC was performed via sublingual SDF imaging using the Microscan device (Microvision Medical, Amsterdam, The Netherlands). This is an established bedside technique for automatic, real-time analysis of MC [15,16,17]. In brief, green light with a wavelength of 530 nm illuminates the sublingual mucosa. Hemoglobin absorbs this wavelength, which makes erythrocytes appear as dark cells moving through the visualized blood vessels. The blood flow in the visualized vessels (field of view: 1044 × 758 µm) is documented as a short video sequence and stored as an AVI-file. This measurement was performed on each day at three different sites of the sublingual mucosa. The quantitative analysis of MC was performed completely automatically using the AVA 4.0 software (Microvision Medical, Amsterdam, The Netherlands). This warrants a high reproducibility with a lower interobserver variability than semi-automatic or manual approaches. Blood vessels with a diameter up to 100 µm were included in the calculation. The measured parameters of MC were number of vessel crossings (NC), number of perfused vessel crossings (PNC), proportion of perfused vessels (PPV), total vessel density (TVD) and perfused vessel density (PVD).

### 2.4. Statistical Analyses

Data are presented as mean ± standard deviation for normally distributed continuous variables and median (interquartile range) for non-normally distributed continuous variables. Continuous MC parameters were non-normally distributed and therefore assessed by the Mann–Whitney U test. The Shapiro–Wilk test was used to test for normal distribution. The serial measured parameters of MC in all three groups were assessed by the Kruskal–Wallis test due to their non-normal distribution. Categorical variables are presented as frequencies and percentages. The comparison of categorical variables was performed by Fisher’s exact test due to the relatively small number of investigated cases. All statistical analyses were performed using SPSS (version 22.0; IBM, Armonk, NY, USA). A two-sided probability-value (*p*) less than 0.05 was considered statistically significant.

## 3. Results

### 3.1. Baseline Clinical Characteristics

The baseline clinical characteristics of TTS and MI patients are presented in Table 1. The cohort consisted of elderly subjects with a majority of female patients. The cardiovascular risk factors, diabetes mellitus (*p* = 0.007), hypercholesterolemia (*p* = 0.006) and smoking (*p* = 0.049), were more frequent in the MI group. Moreover, patients in the MI group had a higher body mass index (*p* = 0.007). TTS patients showed a longer hospital stay (*p* = 0.001) and a more distinctive reduction in left ventricular ejection fraction (LVEF) (*p* = 0.009). In contrast to MI patients, TTS patients showed a complete recovery of LVEF after the follow-up period (*p* = 0.002). During follow-up, two TTS patients died (both due to septic shock). Three TTS and six MI patients were lost to follow-up.

### 3.2. Assessment of Systemic Microcirculation

The results of the MC measurements are summarized in Table 2. The TTS group showed a slightly higher NC and PNC on day 1 [NC: MI–TTS (*p* = 0.004); PNC: Control–TTS (*p* = 0.004), MI–TTS (*p* = 0.009)]. On day 3, TTS patients showed a minimally higher PNC compared to the control group (*p* = 0.007). The control group had a lower PPV [Control–MI (*p* < 0.001), TTS–MI (*p* = 0.008)]. Day 5 presented a higher PPV and PVD in TTS patients compared to healthy controls [PPV: Control–TTS (*p* = 0.013), PVD: Control–TTS (*p* = 0.039)]. The MC parameters after 3 months did not show any significant difference between the investigated groups. The MC parameters in the MI group showed slightly elevated values during follow up. However, these differences were not statistically significant.

The chronological sequences of the MC results are illustrated in Table 3 for TTS and Table 4 for MI. The assessed MC parameters did not show any relevant changes over time in the TTS group. The situation is almost the same in the MI group, besides a marginally higher PPV on day 3 compared to day 1 (*p* = 0.008).

**Figure 2:**
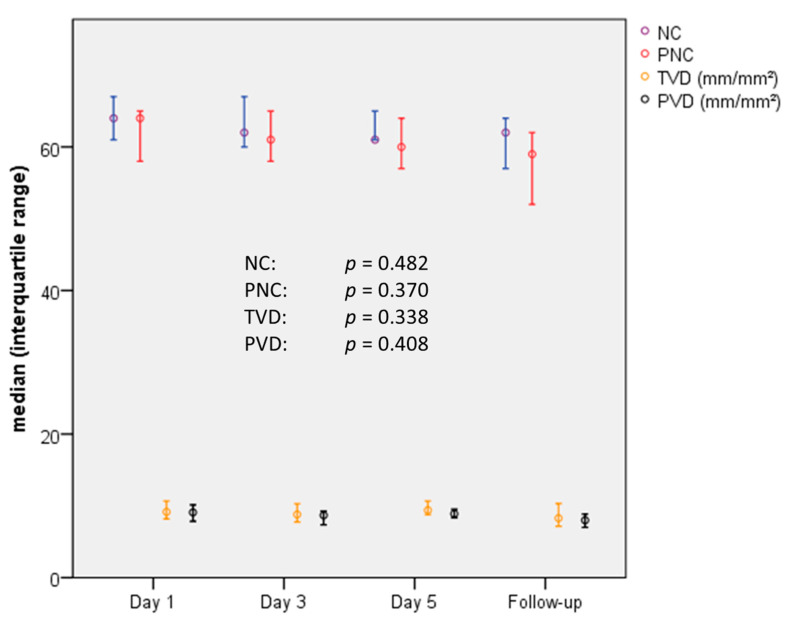
Chronological sequence of MC parameters in TTS patients: The measured parameters did not show any significant change during the observation period. Values are median (interquartile range). MC = microcirculation, NC = number of vessel crossings, PNC = number of perfused vessel crossings, TVD = total vessel density, PVD = perfused vessel density.

The overall view on the assessed parameters shows that there are only minimal differences between the investigated groups. Especially in the TTS group, the assessed parameters did not show any relevant changes during the temporal course (Figure 2).

## 4. Discussion

To the best of our knowledge, the present study is the first that comprehensively investigates systemic microvascular dysfunction by SDF in TTS patients. The main findings of our study can be summarized as follows: (1) the investigated MC parameters of TTS and MI patients did not show major differences compared to healthy individuals; (2) TTS patients did not show any relevant changes in microvascular function during the observation period. Consequently, we could not identify a systemic microvascular dysfunction as a contributing factor for the development of TTS.

It is generally accepted that the activation of the sympathetic nervous system with or without a preceding emotional or physical trigger is a cornerstone of the pathophysiological concept of TTS. However, the exact link between sympathetic overdrive and myocardial dysfunction is still missing. Recent data suggest that a transient microvascular and endothelial dysfunction might induce a contractile dysfunction of the affected myocardium [6,8]. Several reports indicate a coronary microvascular dysfunction during the acute phase of TTS as demonstrated by abnormal findings of myocardial contrast echocardiography [18], a prolonged thrombolysis in myocardial infarction frame count [19] and a reduced coronary flow reserve [20,21,22]. This is in line with the observation of Galiuto et al., who induced a transient improvement in myocardial contractility by intravenous administration of adenosine in patients with TTS [23]. These findings support the hypothesis that microvascular dysfunction is part of the pathogenesis of TTS—or at least a contributing factor. However, it is unclear if microvascular dysfunction is restricted to the coronary vessels or is a systemic phenomenon with cardiac manifestation. We assumed that an overactive sympathetic nervous system might lead to a systemic microvascular dysfunction, which would, therefore, be detectable via the SDF imaging of the sublingual mucosa. This hypothesis was supported by the presence of elevated plasma levels of catecholamines [10] and endothelin-1 [11] during the acute phase of TTS; both are surrogate parameters for a systemic microvascular vasoconstriction. SDF imaging is a well-established method for the investigation of microvascular disturbances during cardiovascular diseases [24,25]. In particular, the ability to monitor the microvascular function of critically ill patients during different states of disease has been shown before [26]. However, we neither found any evidence for an impairment in the systemic microvascular function in the current study, nor could we prove relevant changes in the systemic MC parameters during the temporal course in patients with TTS. Some MC parameters occasionally showed minor differences between the investigated groups (especially higher MC parameters in the TTS group). The absolute values were small and the affected parameters changed over time. Therefore, we interpret this finding as a random effect. A pathophysiologic pattern is unlikely. The situation is similar with the MC parameters of the MI group during follow up. They showed slightly higher values. However, these differences were not statistically significant. These results are in line with the observations of Madhavan et al. who could not confirm elevated catecholamine levels in the systemic circulation and documented normal plasma levels in patients with TTS [27]. On the other hand, Kume et al. detected markedly increased plasma levels of norepinephrine in blood samples from the coronary sinus, suggesting a local catecholamine excess [28]. Moreover, it has already been shown that the microvascular function differs depending on the investigated microvascular bed. In an animal model of septic shock, a normalized sublingual MC coexisted with a severely impaired intestinal microvascular function [29]. Therefore, our findings show that a possible underlying microvascular dysfunction is not a systemic phenomenon but might rather be a local effect on the level of the coronary microvascular bed.

Several limitations of this study need to be acknowledged. The number of investigated patients is small and the follow-up rate after 3 months was lower than expected. Second, we used microcirculatory perfusion parameters that were software derived. Third, the sublingual approach does not take into account perfusion heterogeneity, which may be increased in disease states [29]. Finally, microvascular flow has not been correlated with other parameters of MC, e.g., biomarkers or local cardiac MC.

## 5. Conclusions

In conclusion, a systemic microvascular dysfunction could not be identified as a contributing factor in the pathogenesis of TTS. Therefore, possible microvascular dysfunction might rather be a local effect, restricted to the coronary microvascular bed.

## Figures and Tables

**Table 1:** Baseline clinical characteristics.

Variable	TTS (*n* = 22)	MI (*n* = 20)	*p*	Control (*n* = 20)
Age, years, mean (SD)	72.3 (12.5)	66.1 (11.3)	0.098	28.8 (6.6)
Female sex, n/N (%)	17/22 (77)	13/20 (65)	0.499	12/20 (60)
Cardiovascular risk factors				
Hypertension, n/N (%)	13/22 (59)	14/20 (70)	0.531	-
Diabetes mellitus, n/N (%)	0/22 (0)	6/20 (30)	**0.007**	-
Hypercholesterolemia, n/N (%)	2/22 (9)	10/20 (50)	**0.006**	-
Current smoking, n/N (%)	4/22 (18)	10/20 (50)	**0.049**	-
Body mass index, kg/m^2^, mean (SD)	23.3 (2.9)	26.3 (3.7)	**0.007**	-
Days of hospitalization, median (IQR)	9.5 (6.8; 11.0)	4.0 (4.0; 8.3)	**0.001**	
Stressful event, n/N (%)	11/22 (50)	-	-	
Emotional, n/N (%)	7/22 (31.8)	-	-	-
Physical, n/N (%)	6/22 (27.3)	-	-	-
Ballooning pattern				
Apical, n/N (%)	14/22 (63.6)	-	-	-
Midventricular, n/N (%)	7/22 (31.8)	-	-	-
Basal, n/N (%)	1/22 (4.5)	-	-	-
Number of diseased vessels				
1, n/N (%)	-	6/20 (30.0)	-	-
2, n/N (%)	-	8/20 (40.0)	-	-
3, n/N (%)	-	6/20 (30.0)	-	-
Infarct related vessel				
Left anterior descending, n/N (%)	-	12/20 (60.0)	-	-
Right coronary artery, n/N (%)	-	6/20 (30.0)	-	-
Left circumflex, n/N (%)	-	2/20 (10.0)	-	-
Initial LV ejection fraction, %, mean (SD)	41.1 (10.4)	49.8 (10.0)	**0.009**	-
Follow-up LV ejection fraction, %, mean (SD)	58.2 (5.4)	49.3 (9.3)	**0.002**	-
CK at admission, U/L, median (IQR)	167.5 (79.5; 340.3)	153.0 (97.0; 610.0)	0.583	-
Troponin T at admission, ng/L, median (IQR)	216.0 (69.9; 618.5)	193.0 (65.0; 1785.0)	0.734	-
Death during follow up, n/N (%)	2/22 (9.1)	0/20 (0.0)	0.489	-

Statistically significant values (*p* < 0.05) are highlighted in bold. CK = creatine kinase, IQR = interquartile range, LV = left ventricular, MI = myocardial infarction, n/N = partial quantity/total quantity, SD = standard deviation, TTS = Takotsubo syndrome.

**Table 2:** Results of microcirculation measurements.

Date	Variable	TTS (*n* = 22)	MI (*n* = 20)	Control (*n* = 20)	*p*
Day 1	NC	64.0 (60.3; 67.8)	57.5 (54.8; 62.0)	61.6 (54.8; 64.4)	**0.014**
	PNC	64.0 (57.3; 65.8)	55.0 (53.0; 59.0)	55.1 (50.9; 60.8)	**0.006**
	PPV (%)	99.12 (94.98; 99.97)	97.69 (93.16; 99.67)	96.30 (85.74; 99.96)	0.268
	TVD (mm/mm^2^)	9.15 (7.93; 11.38)	7.42 (6.50; 10.04)	8.08 (7.39; 10.36)	0.134
	PVD (mm/mm^2^)	9.09 (7.79; 11.11)	7.29 (6.32; 9.78)	7.31 (7.08; 9.24)	0.053
Day 3	NC	62.0 (59.8; 67.0)	61.0 (57.0; 63.0)	61.6 (54.8; 64.4)	0.198
	PNC	61.0 (58.0; 65;3)	59.5 (56.5; 62.8)	55.1 (50.9; 60.8)	**0.022**
	PPV (%)	98.96 (95.08; 99.97)	99.99 (99.91; 100.00)	96.30 (85.74; 99.96)	**0.001**
	TVD (mm/mm^2^)	8.80 (7.71; 10.42)	8.84 (7.19; 10.49)	8.08 (7.39; 10.36)	0.700
	PVD (mm/mm^2^)	8.69 (7.28; 9.34)	8.84 (7.17; 10.06)	7.31 (7.08; 9.24)	0.323
Day 5	NC	61.0 (60.5; 66.0)	58.0 (51.5; 63.5)	61.6 (54.8; 64.4)	0.175
	PNC	60.0 (57.0; 54.5)	53.0 (47.0; 61.0)	55.1 (50.9; 60.8)	**0.018**
	PPV (%)	98.98 (94.02; 99.88)	97.08 (84.92; 99.98)	96.30 (85.74; 99.96)	0.691
	TVD (mm/mm^2^)	9.39 (8.65; 10.84)	7.67 (6.52; 9.86)	8.08 (7.39; 10.36)	0.052
	PVD (mm/mm^2^)	8.89 (8.33; 9.84)	7.67 (5.77; 8.56)	7.31 (7.08; 9.24)	**0.045**
Follow-up	NC	62.0 (56.5; 65.0)	63.5 (57.8; 66.0)	61.6 (54.8; 64.4)	0.407
	PNC	59.0 (52.0; 63.0)	62.0 (56.8; 66.0)	55.1 (50.9; 60.8)	0.075
	PPV (%)	95.74 (89.87; 99.56)	99.60 (97.88; 99.97)	96.30 (85.74; 99.96)	0.248
	TVD (mm/mm^2^)	8.29 (7.12; 10.79)	9.00 (7.30; 9.90)	8.08 (7.39; 10.36)	0.813
	PVD (mm/mm^2^)	8.00 (6.84; 9.27)	8.60 (6.94; 9.82)	7.31 (7.08; 9.24)	0.513

Values are median (interquartile range). Statistically significant values (*p* < 0.05) are highlighted in bold. FUP = follow-up after 3 months, NC = number of vessel crossings, PNC = number of perfused vessel crossings, PPV = proportion of perfused vessels, TVD = total vessel density, PVD = perfused vessel density. Day 1: NC: MI–TTS (*p* = 0.004); PNC: Control–TTS (*p* = 0.004); MI–TTS (*p* = 0.009). Day 3: PNC: Control–TTS (*p* = 0.007); PPV: Control–MI (*p* < 0.001); TTS–MI (*p* = 0.008). Day 5: PNC: Control-TTS (*p* = 0.013); PVD: Control–TTS (*p* = 0.039).

**Table 3:** Chronological sequence of MC parameters in TTS patients.

	Day 1	Day 3	Day 5	FUP	*p*
NC	64.0 (60.3; 67.8)	62.0 (59.8; 67.0)	61.0 (60.5; 66.0)	62.0 (56.5; 65.0)	0.482
PNC	64.0 (57.3; 65.8)	61.0 (58.0; 65;3)	60.0 (57.0; 54.5)	59.0 (52.0; 63.0)	0.370
PPV (%)	99.12 (94.98; 99.97)	98.96 (95.08; 99.97)	98.98 (94.02; 99.88)	95.74 (89.87; 99.56)	0.507
TVD (mm/mm^2^)	9.15 (7.93; 11.38)	8.80 (7.71; 10.42)	9.39 (8.65; 10.84)	8.29 (7.12; 10.79)	0.338
PVD (mm/mm^2^)	9.09 (7.79; 11.11)	8.69 (7.28; 9.34)	8.89 (8.33; 9.84)	8.00 (6.84; 9.27)	0.408

Values are median (interquartile range). FUP = follow-up after 3 months, MC = microcirculation, NC = number of vessel crossings, PNC = number of perfused vessel crossings, PPV = proportion of perfused vessels, TVD = total vessel density, TTS = Takotsubo syndrome, PVD = perfused vessel density.

**Table 4:** Chronological sequence of MC parameters in MI patients.

	Day 1	Day 3	Day 5	FUP	*p*
NC	57.5 (54.8; 62.0)	61.0 (57.0; 63.0)	58.0 (51.5; 63.5)	63.5 (57.8; 66.0)	0.095
PNC	55.0 (53.0; 59.0)	59.5 (56.5; 62.8)	53.0 (47.0; 61.0)	62.0 (56.8; 66.0)	0.054
PPV (%) *	97.69 (93.16; 99.67)	99.99 (99.91; 100.00)	97.08 (84.92; 99.98)	99.60 (97.88; 99.97)	**0.008**
TVD (mm/mm^2^)	7.42 (6.50; 10.04)	8.84 (7.19; 10.49)	7.67 (6.52; 9.86)	9.00 (7.30; 9.90)	0.738
PVD (mm/mm^2^)	7.29 (6.32; 9.78)	8.84 (7.17; 10.06)	7.67 (5.77; 8.56)	8.60 (6.94; 9.82)	0.459

Values are median (interquartile range). Statistically significant values (*p* < 0.05) are highlighted in bold. FUP = follow-up after 3 months, MC = microcirculation, MI = myocardial infarction, NC = number of vessel crossings, PNC = number of perfused vessel crossings, PPV = proportion of perfused vessels, TVD = total vessel density, PVD = perfused vessel density. * Days 1–3 (*p* = 0.001).

## Data Availability

The data presented in this study are available on request from the corresponding author.
